# Major Histocompatibility Complex and Malaria: Focus on *Plasmodium vivax* Infection

**DOI:** 10.3389/fimmu.2016.00013

**Published:** 2016-01-27

**Authors:** Josué da Costa Lima-Junior, Lilian Rose Pratt-Riccio

**Affiliations:** ^1^Laboratory of Immunoparasitology, Oswaldo Cruz Institute – Fiocruz, Rio de Janeiro, Brazil; ^2^Laboratory of Malaria Research, Oswaldo Cruz Institute – Fiocruz, Rio de Janeiro, Brazil

**Keywords:** malaria, *P.vivax*, MHC, HLA, vaccine

## Abstract

The importance of host and parasite genetic factors in malaria resistance or susceptibility has been investigated since the middle of the last century. Nowadays, of all diseases that affect man, malaria still plays one of the highest levels of selective pressure on human genome. Susceptibility to malaria depends on exposure profile, epidemiological characteristics, and several components of the innate and adaptive immune system that influences the quality of the immune response generated during the *Plasmodium* lifecycle in the vertebrate host. But it is well known that the parasite’s enormous capacity of genetic variation in conjunction with the host genetics polymorphism is also associated with a wide spectrum of susceptibility degrees to complicated or severe forms of the disease. In this scenario, variations in genes of the major histocompatibility complex (MHC) associated with host resistance or susceptibility to malaria have been identified and used as markers in host–pathogen interaction studies, mainly those evaluating the impact on the immune response, acquisition of resistance, or increased susceptibility to infection or vulnerability to disease. However, due to the intense selective pressure, number of cases, and mortality rates, the majority of the reported associations reported concerned *Plasmodium falciparum* malaria. Studies on the MHC polymorphism and its association with *Plasmodium vivax*, which is the most widespread *Plasmodium* and the most prevalent species outside the African continent, are less frequent but equally important. Despite punctual contributions, there are accumulated evidences of human genetic control in *P. vivax* infection and disease. Herein, we review the current knowledge in the field of MHC and derived molecules (HLA Class I, Class II, TNF-α, LTA, BAT1, and CTL4) regarding *P. vivax* malaria. We discuss particularly the results of *P. vivax* studies on HLA class I and II polymorphisms in relation to host susceptibility, naturally acquired immune response against specific antigens and the implication of this knowledge to overcome the parasite immune evasion. Finally, the potential impact of such polymorphisms on the development of vaccine candidate antigens against *P. vivax* will be studied.

## Introduction

Caused by blood-borne apicomplexan parasites of the genus *Plasmodium*, malaria remains a major public health problem. Malaria transmission occurs in 96 countries and territories, and according to the latest estimates, 3.3 billion people are at risk of infection. Approximately 214 million cases and 438,000 deaths due to malaria occur worldwide, mainly of children under 5 years. The great majority of cases (88%) and deaths (90%) occurs in Africa, followed by Southeast Asia (10%) and Eastern Mediterranean region (2%) (1). Of the five *Plasmodium* species that affect humans, *Plasmodium vivax* is responsible for about 6% of the world estimated cases. However, outside sub-Saharan Africa, *P. vivax* accounts for 51% of all malaria cases, being the most widespread *Plasmodium* species (1).

*Plasmodium* parasites have a complex lifecycle, which includes the development of a sexual cycle in the invertebrate vector, the female of the *Anopheles* mosquitos, and an asexual cycle in the vertebrate hosts. Infection with *Plasmodium* parasites presents an asymptomatic stage, pre-erythrocytic, which occurs in the liver followed by a symptomatic erythrocytic stage, when merozoites arisen during pre-erythrocytic stage invade red blood cells. The rupture of the erythrocytic schizont is typically accompanied by clinical symptoms, because of the release of parasite derived toxins, such as phospholipids, that can activate immune cells leading to the production of inflammatory cytokines that can, directly or indirectly, contribute to the elimination of the parasite and complications associated with infection.

At first, humans are susceptible to malaria, even those who have already contracted the disease several times. However, young children, pregnant women, and adults from non-endemic areas are particularly susceptible to develop severe malaria. In high endemic malaria areas, with repeated exposure, older children and adults develop considerable degree of protection from death and severe malaria, and thus, the clinical manifestations are milder, or even absent, although sterile immunity is probably never achieved and the infected immune individuals continue to present parasites in the blood for long periods, probably in the presence of very mild symptomatology (2, 3). It has been proposed that these changes reflect the parasitological and clinical immunity collectively referred to as naturally acquired immunity, which generally determines not only the age-specific incidence and prevalence of infections but also the expression of pathological processes that underlie the clinical manifestations of infection.

The spectrum of malaria clinical manifestations generally differs between adults and children and from person to person, ranging from asymptomatic infection to clinical symptoms as fever, nausea, headache, and muscle pain, chills and vomiting and, in 1–2% of the cases, to severe malaria, leading to multiorgan system involvement, severe anemia, and death (4–6). *Plasmodium falciparum* is the most virulent agent and responsible for the majority of severe malaria deaths (1). Severe malaria due to *P. falciparum* may present as confusion, drowsiness, excitement, convulsions, delirium, and coma. The differences observed in the clinical forms of the disease as well as the underlying pathophysiological processes are still under investigation, but it is now clear that the genetic factors influence the spectrum of clinical manifestations and the evolution and severity of the disease (7, 8).

The classical framework of the influence of genetic factors in malaria evolution and severity is the protective effect of certain hemoglobinopathies. The first observations were postulated in the late 40s by Haldane, known as one of the three founders of population genetics and acknowledged as the first person to suggest that disease could be an important evolutionary force in humans (9). Based on the distribution of thalassemia in the Mediterranean, Haldane proposed that certain hemoglobinopathies are highly prevalent in regions where malaria is endemic due to the protection against the severe disease (10). According to the Haldane’s malaria hypothesis, this could result in a “balanced polymorphism” where the homozygote disadvantage for inherited erythrocyte disease is compensated through the resistance of the heterozygote where malaria is endemic (11–14). Thus, it has been proposed that malaria is associated to gene selective pressure in the human genome, and it has been associated with some genetic diseases. After that, several reports have shown that genetic disorders, such as thalassemias, sickle-cell trait, glucose-6-phosphate dehydrogenase (G6PD) deficiency, ovalocytosis, Hemoglobin (Hb) S, HbC, HbE, and complement receptor-1 (CR1) deficiency, are associated with malaria susceptibility or resistance. Case–control studies have shown that these polymorphisms reduce the risk of severe and complicated malaria. Among the mechanisms involved in the protection against *P. falciparum* severe malaria are reduced invasion of erythrocyte by the parasite, decreased intracellular parasite growth, increased phagocytosis, and enhanced immune response against parasite-infected erythrocyte (14–17). Besides these genetic disorders, other polymorphisms in genes encoding the immune system molecules may also be involved in malaria outcome (Table 1).

**Table 1:** **Genetic polymorphisms of the vertebrate host and associations with the natural resistance to malaria**.

Genetically based resistance mechanisms		Gene/locus	Function	Phenotype	Reference
Hemoglobinophaties	α-Thalassemia	HBA (16p13.3)	Hemoglobin composition	Protection against severe malaria	(18–20)
	β-Thalassemia	HBB (11p15.5)	Hemoglobin composition	Protection against severe malaria	(11, 21)
	Sickle cell disease	HBB (11p15.5)	Hemoglobin composition	Protection against severe malaria	(19, 22, 23)
	Hemoglobin C (HbC)	HBB (11p15.5)	Hemoglobin composition	Reduced risk of severe and non-severe *P. falciparum* infections	(24, 25)
	Hemoglobin E (HbE)	HBB (11p15.5)	Hemoglobin composition	Protection against severe malaria and high parasitemia	(26, 27)
Enzymes	Glucose-6-phosphate dehydrogenase (G6PD)	G6PD (Xq28)	Protection of erythrocyte against oxidative stress	Resistance against *P. falciparum* infection and severe malaria	(16, 28–30)
	Pyruvate kinase (PK)	PKLR (1q21)	Erythrocyte metabolism	Protection against *P. falciparum* infection	(31)
Erythrocyte	Ovalocytosis	SLC4A1 (17q21-22)	Anion exchanger	Protection against severe malaria by *P. falciparum* and reduced risk of *P. vivax* infection	(32–34)
	Duffy antigen	ACKR1/FY (1q21-q22)	Chemokine receptor	Decreased risk/resistance of *P. vivax* infection	(35–38)
Immunogenetic variants	Human leukocyte antigens (HLA)	HLA (6p21.3)	Component of the immune system	Protection against severe malaria and antiplasmodial immune response	(39–44)
	Complement component (3b/4b) receptor 1 (CR1)	CR1 (1q32)	Removing immune complexes/cytoadherence	Protection against severe malaria	(45)
	Nitric oxide synthase 2 r	NOS2A (17q11.2)	Nitric oxide production	Protection against severe malaria	(46, 47)
	Tumor necrosis factor (TNF)	TNF (6p21.3)	Proinflamatory activities	Severe malaria	(48–51)
	Interferon gamma (IFN)	IFNG (12q14)	Proinflamatory activities	Reduced risk to develop severe malaria	(52)
	Interleukin 4 (IL4)	IL4 (5q31.1)	Anti-inflammatory activities	Antimalarial antibody levels and reduced risk to develop severe malaria	(53, 54)
	Interleukin 10 (IL10)	IL10 (1q31-q32)	Regulation of the immune response	Reduced risk to develop severe malaria and anemia	(55, 56)

Considering the intense selective pressure, the number of cases and the mortality rates associated with *P. falciparum* infection, specific studies of association between genetic factors and *P. vivax* are less frequent, even though this species is the most widespread *Plasmodium*, the most prevalent species outside the African continent, and with increasing evidences of associated death (57, 58). The observation that *P. vivax* malaria is rare in West Africa and that most sub-Saharan Africans are negative to blood group Duffy was the first evidence regarding *P. vivax* natural resistance. It led to the discovery that *P. vivax* uses the Duffy blood group antigen as a receptor to invade erythrocytes (59). Populations with the null phenotype of Duffy, although susceptible to the hepatic malaria stage, are less susceptible to *P. vivax* merozoite invasion. Moreover, there are accumulated evidences of the relationship between immune response to *P. vivax* antigens and major histocompatibility complex (MHC) genes. Therefore, in the present study, we review the current knowledge in the field of MHC molecules regarding *P. vivax* malaria.

## *P. vivax* Malaria and the Immune System

**Figure 1:**
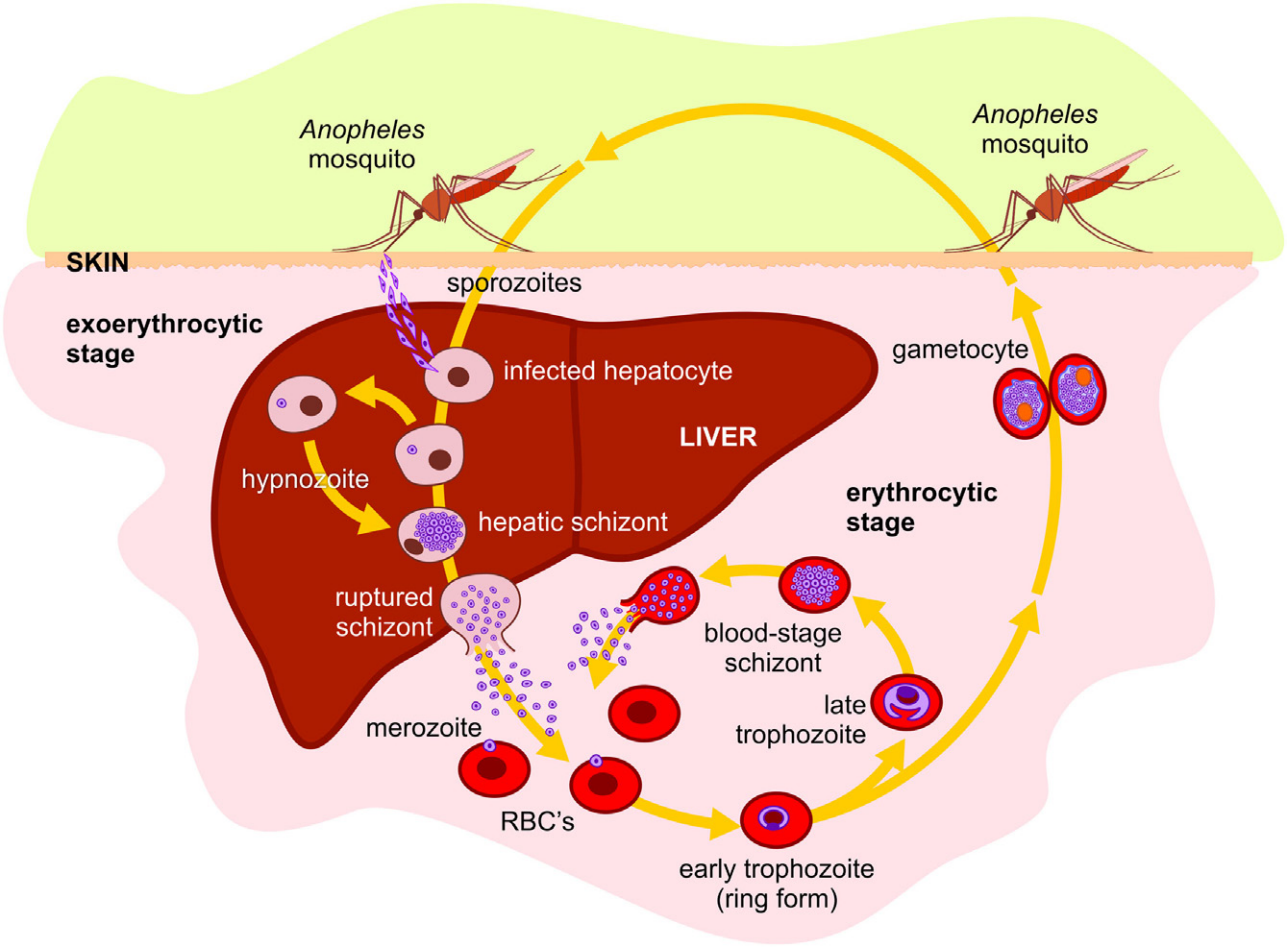
***Plasmodium vivax* lifecycle in human host: *P. vivax* is transmitted to humans by the bite of an infected female of the *Anopheles* mosquito, releasing the salivary fluid carrying sporozoites into the tissues or directly into the bloodstream**. From the tissues, the motile sporozoites can penetrate small blood vessels. In hepatic sinusoids, they penetrate through Kupffer cells into Space of Disse and invade hepatocytes to begin the exo-erythrocytic or liver-stage cycle. The sporozoite differentiates into mature liver-stage schizont with thousands of uninucleated merozoites surrounded by a parasitophorous membrane. The hepatocyte containing mature liver schizonts ruptures releasing merosomes. These merosomes are transported into the general blood circulation and break, releasing merozoites which invade young red blood cells (reticulocytes), beginning the erythrocytic or blood-stage cycle. *P. vivax* has dormant liver hypnozoite stages, which can reactivate and lead to blood-stage relapses. Within the erythrocyte, the merozoite differentiates in erythrocytic trophozoite. When fully mature, the infected erythrocyte ruptures, releasing the merozoites, which then invade new erythrocytes, initiating the entire intraerythrocytic-stage cycle, rupture, and reinvasion. Alternatively, some merozoites can develop gametocytes. During blood feeding, female mosquito of a susceptible *Anopheles* species can ingest the gametocytes, beginning the sexual stage of the life cycle. In the midgut of the mosquito, gametocytes escape from erythrocytes and become sexually stimulated. The male gamete fuses with the female, forming a diploid zygote. Therefore, the zygote is transformed into an invasive parasite stage ookinete. The ookinete traverses the midgut wall by passing through epithelial cells and comes to rest adjacent to the basal lamina where it transforms into an oocyst that undergoes multiple nuclear divisions producing several thousand sporozoites. At maturity, the oocyst breaks open and the sporozoites are released into the hemocele of the mosquito, migrating and penetrating the salivary glands. In the salivary glands, the sporozoites become infectious to humans, completing the life cycle.

Like in other species of the *Plasmodium* genus, *P. vivax* lifecycle is a complex process and requires an invertebrate and a vertebrate host for survival and perpetuation (Figure 1). Therefore, during its entire life cycle in humans, *P. vivax* undergoes multiple morphological and antigenically distinct stages and can be attacked by different immune mechanisms, depending on the stage and whether the parasite is within or outside the host cell. During the migration through the bloodstream to the liver, antibodies can block sporozoite migration and/or invasion of hepatocytes, repressing lifecycle progression (60–63). In the liver stage, infected hepatocytes are potential targets of CD4^+^ and CD8^+^ T cells, although the immune response mediated by NK cells and T gamma-delta T cells also participates in the immune response against pre-erythrocytic forms stimulating other cell populations secreting cytokines or acting directly on the infected hepatocyte (64–66). After being released from merosomes, free merozoites are susceptible to host immune responses. Merozoites can be the target of opsonizing antibodies, triggering cell-mediated merozoite killing or blocking merozoite proteins responsible for the initial interaction with the molecules on the surface of erythrocytes, preventing invasion (67, 68). Considering that the MHCs Class I and II antigens are absent on the surface of the erythrocytes, the immune response against blood stage forms involves mainly antibodies. During the intraerythrocytic stage, antibodies may coalesce merozoites at or just before the rupture of erythrocytes, preventing their release and spread into the bloodstream, essential for the clearance of parasitemia in the later stages of the infection (2, 67, 68). Although antibodies have a critical role in the development of immunity against erythrocytic forms, studies indicate that the development of the immune response also involves monocytes, neutrophils, CD4^+^ T cells, NK cells, and NKT cells (68).

Overall, one may say that the cellular immune response is more important in the control of the hepatic forms of the parasite, whereas the humoral immune response seems to be more important to the control of its erythrocytic stage. Since *P. vivax* stimulates various components of the immune system, the balance of this activation can represent a fine line between inhibition of the parasite growth and immunopathology. Thus, it is acceptable to consider that polymorphisms in genes encoding immune system molecules, especially those located at MHC locus, could be involved in *P. vivax* malaria outcome.

## HLA Genetic Region

**Figure 2:**
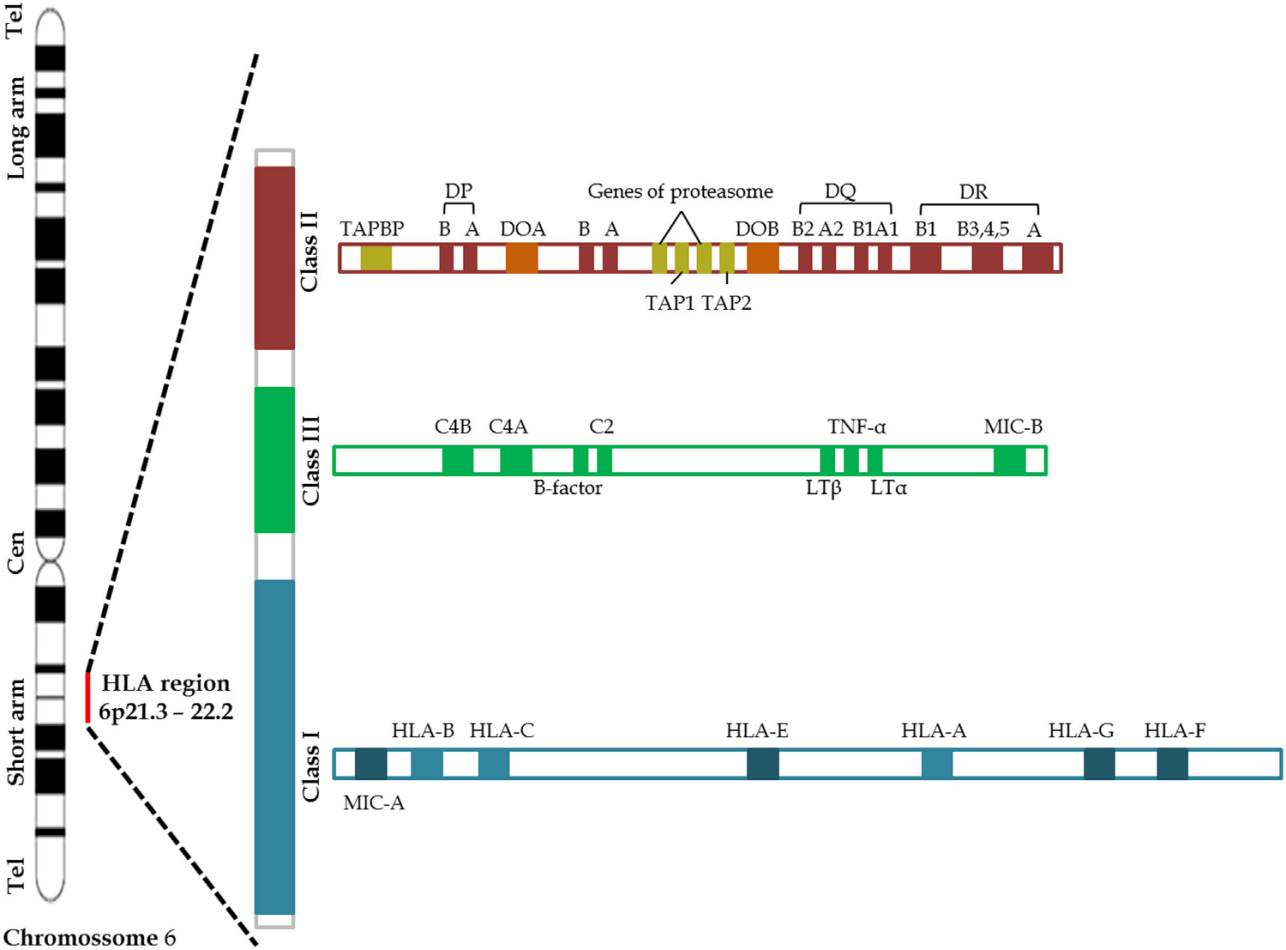
**Schematic representation of the human Chromosome 6 including the main MHC genes** (69).

The MHC, referred as human leukocyte antigen (HLA) system in humans, is an extremely polymorphic region encoding for the major molecules in charge of antigen presentation on the cell surface, and it has been one of the most intensively studied areas in the human genome (69, 70). Located in the short arm of the chromosome 6, HLA complex consists of more than 200 genes categorized into three basic groups: class I, class II, and class III (Figure 2). Class I molecule is a heterodimer consisting of a heavy chain and a light chain, the beta-2 microglobulin. HLA Class I genetic region encodes the heavy chain of the classics HLA-A, -B, and -C molecules, besides HLA-E, -F, -G, and the MHC class I polypeptide-related sequence A (MICA) and MICB. Class I molecules are expressed in nearly all cells and play a central role in the immune system by presenting peptides derived from the endoplasmic reticulum lumen. Class II molecules are heterodimers formed by α and β chains. HLA class II genetic region, initially called Immune response (Ir) genes due to its role in controlling the immune response, encodes the α and β chains of the HLA-DR, -DQ, -DP, -DM, and -DO molecules and peptide transporter proteins (TAP) 1 and (TAP) 2 (69–71). Class II molecules are predominantly expressed on antigen-presenting cells (APC), such as macrophages, dendritic cells, B cells, Langerhans cells, and Kupffer cells, although some cells may express class II molecules during inflammatory process (70, 72, 73). The proteins produced from HLA class III genes have somewhat different functions, some of which involve participation in inflammation processes and other immune system activities. HLA-Class III genetic region encodes C2 and C4 complement components and tumor necrosis factor (TNF) superfamily (70). The functions of some HLA genes are unknown.

The polymorphism of HLA has been useful in the search of donors with compatible grafts in tracing population migration as well as in its potential relationship to pathogen-mediated selection. Further, assessing and comparing the polymorphism of HLA allows to better define the extent of the genetic variability in humans as well as the reasons of this diversity. The HLA region is associated with more diseases (mainly autoimmune and infectious diseases) than any other region of the genome (74–78).

## HLA Class I and II Genes and *P. vivax* Antigens

The importance of HLA genes influencing malaria outcome has been demonstrated since studies conducted by Hill and colleagues who elegantly illustrated the influence of HLA genes in the protection against an intracellular pathogen and how the polymorphism of HLA genes may have evolved through selection of molecules induced by the pathogen. In a study of over 2000 children in West Africa, Hill et al. showed that carriers of HLA Class I Bw53 and HLA class II DRB1*1302-DQB1*0501, frequently occurring in sub-Saharan Africa, were protected against severe malaria (39). Later studies showed that HLA-B53 restricted cytotoxic T cells recognize peptides corresponding to regions of *P. falciparum* Liver Stage Antigen-1 (79). Thus, HLA molecules have been used as genetic markers in an attempt to determine the presence of genetic modulation of the immune response during malaria infection. Considering the increasing focus on the development of subunit malaria vaccines, studies on the influence of HLA molecules in the immune response in ethnically diverse populations are important before the implementation of vaccine trials. This is particularly relevant for *P. vivax*, which affects populations with high diversity of genetic backgrounds.

In this scenario, the circumsporozoite surface protein (CSP) is the most abundant polypeptide present in the sporozoite covering. This protein is involved in the motility and invasion of the sporozoite during its entrance in the hepatocyte (80). The *csp* gene encodes a protein, which has in its central portion, a highly immunogenic repetitive region. Based on the *csp* gene, two variants, VK247 and *P. vivax*-like, have been described. They differ from the classical form (VK210) by sequence variations in the central region of the gene (81, 82). A study performed by Oliveira-Ferreira and others with 108 individuals living in Rondonia State, in the Southwestern part of the Brazilian Amazon, observed a significant association between the antibody response to the CSP repeats of VK247and the presence of HLA-DRB1*16 and between the presence of HLA-DRB1*07 and the absence of antibody responses to the CSP repeats of VK210 (83). More recently, Storti-Mello and co-workers described a significant association between the absence of antibody response to the CSP amino-terminal region and the presence of HLA-DRB1*03 and DR5 in a study with 55 individuals from different regions of the Brazilian Amazon (84) (Table 2).

**Table 2:** **Associations between HLA-DRB1 and HLA-DQB1 allelic groups and antibody response to *P. vivax* antigens**.

Antigen/protein	HLA	Association	Reference
CSP VK247 variant	DRB1*16	+	(83)
CSP VK210 variant	DRB1*07	−	(83)
CSP peptide N	DRB1*03	−	(84)
CSP peptide N	DRB1*11, *12 (DR5)	−	(84)
MSP3-NT	DRB1*04	+	(85)
MSP3-CT	DRB1*04	+	(85)
MSP3-CT	DQB1*03	+	(85)
MSP3-CT	DQB1*06	−	(85)
MSP3-FL	DRB1*16	−	(85)
MSP9-RIRII	DRB1*01	−	(85)
MSP9-RIRII	DRB1*04	+	(85)
MSP9-RII	DRB1*01	−	(85)
MSP9-RII	DRB1*04	+	(85)
MSP9-CT	DRB1*04	+	(85)
AMA-1	DRB1*03	+	(84)
RBP-1	DRB1 and DQB1 alleles	Not found	(86)
MSP1-19	DRB1 and DQB1 alleles	Not found	(84, 85)
DBP	DRB1 alleles	Not found	(87)

Merozoite surface proteins (MSPs) have been reported as abundantly expressed on the surface of merozoites and can contribute to the initial recognition of erythrocytes. MSP-1, MSP-3, and MSP-9 have been considered important vaccine candidates based on their location, on their recognition by antibodies from individuals naturally exposed to *P. vivax*, their immunogenic properties in animal models, and evidence of the induction of antibodies able to inhibit parasite-growth (88–95). Therefore, considering the importance of the antibodies against MSPs in the development of anti-parasite immunity, studies have also focused the evaluation of the genetic restriction of the anti-MSP humoral response. Storti-Melo et al. analyzed the influence of the HLA-DRB1 alleles on antibody levels against the amino-terminal region of the MSP-1 in individuals from the Brazilian Amazon and observed significant association between high levels of antibodies for MSP-1 and the presence of HLA-DRB1*03 (84). In contrast, no evidence of a specific HLA-DR or HLA-DQ restriction for the antibody response to MSP-1 was observed in a study carried out by Lima-Junior et al. in 276 individuals living in Rondonia State in the Brazilian Amazon (85). However, in that paper, the authors showed HLA associations with IgG antibody response against different regions of MSP-3 and MSP-9 proteins. A high frequency of responders to carboxy-terminal (CT) and amino-terminal (NT) regions of MSP-3 were defined in HLA-DRB1*04 carriers and to MSP-3CT also defined in HLA-DQB1*03 carriers. Additionally, a high frequency of non-responders to MSP-3CT and the presence of HLA-DQB1*06 and to a recombinant protein representing the full length (FL) of MSP-3 with the presence of the HLA-DRB1*16 allele were observed. Regarding MSP-9, the presence of HLA-DRB1*04 was positively associated with the IgG immune response against all constructions used in the study, the amino-terminal domain (NT) and the C-terminal blocks of tandem repeats (RII and RIRII), while the presence of the HLA-DRB1*01 was associated with the high frequency of non-responders only to the repeated region (Table 2).

However, other studies did not find associations between HLA-DR or HLA-DQ alleles and antibody response to *P. vivax* antigens. In a study performed by Ferreira and co-workers, no genetic restriction mediated by HLA-DRB1* and HLA-DQB1* against two constructions of *P. vivax* Reticulocyte Binding Protein-1 (PvRBP1) was verified in more than 500 HLA alleles from different individuals from communities in the Amazon region of Brazil (86). Moreover, regarding the cellular response, Arevalo-Herrera et al. also did not observe association between HLA and cellular immune response of healthy volunteers vaccinated with CSP derived long synthetic peptides (96) and Lima-junior et al. describe five promiscuous peptides from MSP-9 which also presented no association between HLA-DRB1 alleles and the cellular immune response (97).

## HLA Class III Genes and *P. vivax*

Several genes of the immune system have proved to be important in relation to the susceptibility or resistance to malaria, especially those associated with severe malaria. Therefore, a common strategy is to identify the mutations in such genes and observe their possible association with the disease outcome. Since Kwiatkowski et al. showed that the TNF was associated with the susceptibility to cerebral malaria (98), numerous mutations have already been identified in the promoter of this gene, which can influence on TNF production rate. In one of those vanguard studies, McGuire and colleagues showed that mutation at position -308 of the TNF promoter region is associated with increased risk of death from cerebral malaria in Africa (51). Analysis of other clinical complications experienced in African children with severe malaria also showed that severe anemia due to malaria is associated with the mutation at position -238 suggesting that the clinical manifestations could also be influenced by genetic determinants located near the TNF gene. In fact, the guanine-to-adenine transition at position -308 in the TNF promoter, which defines the rare allele TNF2 is strongly associated with the MHC haplotypes HLA-A1, B8, DR3 and was also reported to influence the TNF promoter activity, enhancing TNF-α production (99). In patients with cerebral, severe malaria and mucocutaneous leishmaniasis, the TNF-α -308G/A polymorphism has been shown to be associated with the outcome and clinical course of the disease (100). However, only in the last years, the influence of these polymorphisms on *P. vivax* infection began to be investigated. On the one hand, in patients with *P. vivax* malaria from India, two single nucleotide polymorphisms (SNP) in the TNF promoter (–308G > A and –1031C > T) were associated with cytokine levels and temperature, but no association related to susceptibility were reported (101). On the other hand, there was neither association between six different TNF SNP polymorphisms and *P. vivax* malaria in Thailand nor differences in allelic distribution among the three distinct ethnic groups assessed by the study: Thai, Burmese, and Karen (102). In Brazil, even TNF-308 GA genotype or A allele carriers presented higher levels of TNF than those with the GG genotype or G allele, no association related to susceptibility was observed in *P. vivax* infected individuals (103, 104). In fact, we tend to reinforce the idea that a SNP is often not sufficient for predicting the susceptibility or resistance of individuals to *P. vivax* malaria. Therefore, the usual approach when investigating the differences in response to malaria infection should be the haplotype analysis. For example, Sortica et al. reported the association of TNF haplotype with a lower susceptibility to *P. vivax* infections, since an uninfected group presented a significantly higher frequency of a specific haplotype (T1031/A863/C857/G308/G238) when compared to *P. vivax* infected individuals (104). However, despite these several evidences of polymorphism in TNF gene in relation to malaria susceptibility in the studies, a larger number of samples and different clinical and epidemiological scenarios are necessary to confirm the associations.

Aside the TNF association studies, the associations between malaria and polymorphisms in other genes located at HLA locus were also the focus of investigations. For example, the nuclear protein HLA-B-associated transcript 1 (BAT1), which is an RNA helicase encoded by the DDX39B gene, has been described as a negative regulator of inflammation by modulating expression of proinflammatory cytokines (such as TNF) (105). Therefore, using mutations in two MHC genes located approximately at 150 kb from each other (TNF and DDX39B) Mendonça et al. reported associations between DDX39B haplotypes and complicated *P. vivax* malaria. Participants with *DDX39B*-22/*DDX39B*-348/*TNF*-308/*IL6*-176 genotype combinations GC/CC/GG/GG and GG/CT/GG/GG had reduced and increased risk, respectively, of developing malaria symptoms (103).

Lastly, other HLA-Class III host candidate gene polymorphisms were also associated with susceptibility/resistance to *Plasmodium* infection. However, the absence of studies using only *P. vivax* infected/exposed individuals makes the definition of genetic polymorphism of HLA-class III genes associated specifically to this species particularly difficult. In *P. falciparum* studies conducted in Africa, a trend of association between LTA polymorphism with antimalarial IgG subclass levels was found but not confirmed by statistical tests (106). Moreover, no LTA polymorphisms were associated with severe malaria in cohorts in Kenya and Malawi in a large study involving >10,000 individuals from three African populations. In Brazilian endemic areas, recently, a study with a large number of candidate gene polymorphisms was performed and the association with susceptibility/resistance to *Plasmodium* infection with clinical (mild) malaria in a population infected with *P. falciparum* or *P. vivax* was investigated. Although no differences between species were found, the results showed, for the first time, an association between alleles of CTL4 gene with malaria, which displayed a significant association with reduced risk for clinical malaria. In addition, two other associations with cytokines were identified, both within MHC class III region, that included TNF and the lymphotoxin alpha (LT-α/LTA) and beta (LT-β/LTB) genes, which are closely related (107).

## HLA and Bioinformatics Applied to *P. vivax* Antigen Discovery

The ultimate goal of MHC binding antigenic peptide prediction is to identify epitopes that activate T-cells and mediate cell-mediated immunity without HLA genotype/haplotype restriction. Recognition of peptide bound to an MHC molecule by a T-cell receptor is a critical step and for T-cell activation binding of peptide to the MHC molecule is a necessary requirement (108). The association of immunogenic fragments (epitopes) to the HLA molecules of class I or II determines what type of cell is to be stimulated and, consequently, what kind of response will be generated. Conventional vaccinology approaches accumulate successes and failures aiming at experimental screening methods to evaluate the presence of HLA restriction in immune response to vaccine candidates. But this conventional process is still laborious, expensive, and time-consuming. Computational prediction methods complement experimental studies, minimize the number of validation experiments, and significantly speed up the epitope mapping process (109). The bioinformatics tools have already e helped identifying promiscuous epitopes within *Leishmania* (110), *Mycobacterium tuberculosis* (111) and HIV (112) antigens. In malaria, epitope identification is particularly challenging, as more than 5000 proteins are encoded by the genome (113, 114), which could generate hundreds of thousands of possible CD4^+^ T cell epitopes. On the other hand, the identification of CD4^+^ and CD8^+^ epitopes from malaria is urgently required to track various vaccine approaches, mainly to evaluate candidates for compositions of subunit vaccines. For example, in *P. falciparum* vaccine research, Doolan et al. first used proteomic approaches to identify 27 highly expressed candidate antigens, and then used HLA-DR binding predictions to identify 723 predicted HLA-DR binders. Of these, 39 peptides binding tightly to HLA-DR variants derived from four newly identified antigenic targets were identified (115). Beyond antigen identification, this application of proteomics and bioinformatics was confirmed as particularly powerful and is likely to prove useful in other applications, particularly as consensus motif prediction approaches.

Despite several T-cell epitopes from pre-erythrocytic (116–118), asexual blood stage (119–121), and gametocyte (122) antigens have been predicted and/or experimentally confirmed for *P. falciparum*, the use of bioinformatics strategies to identify potentially important epitopes in *P. vivax* is still restricted. The majority of the studies focusing on the detection of B or T-cell epitopes have used conventional screening methods (94, 123–125). Only few studies have already used prediction servers to trial the most promising epitopes to be used in validation assays (Table 3). One of the first prediction studies on *P. vivax* reported the results of *in silico* analysis of PvMSP-1 vaccine candidates in relation to potential HLA restricted or promiscuous CD4 and CD8 epitopes (126). More recently, Kumar et al. using several computational screening methods analyzed 10 protein sequences of *P. vivax* proteins, including vaccine candidates, such as MSP-1, MSP-9, Pvs25, and PvS28 in relation to potential antigenicity, promiscuity and binding to several HLA class I and II alleles. The best scored T-CD4 and T-CD8 epitopes for each antigen were also identified (127). Even with promising results, these bioinformatics approach reported is still dependent on experimental validation. In this scenario, our previous studies reported that along all PvMSP-9 N-terminal 11 peptides were highly predicted by the ProPred algorithm to be promiscuous, of which only five of them were recognized at high frequency by PBMCs from individuals living in malaria endemic areas presenting a large variety of HLA class II allelic groups (97). If the conventional screening methods had been applied, at least 40 overlapping peptides should have been synthetized and tested individually in order to select these promiscuous epitopes; on the other hand, if we had used only prediction approaches, five non-immunogenic peptides could have been selected. Although bioinformatics approach has lately accumulated more successes than failures, the confidence level (approximately 50%) for predicting epitopes to MHC class II molecules is far from perfection and in some cases can cause mismatches between predicted versus experimental results. This can happen mainly because these molecules accept a wider range of peptides in size and binding registers (16). For example, two universal epitopes were described in PvDBP sequence using conventional vaccinology experiments; however, the SYFPEITHI-binding prediction for the HLA-DRB1*0101 molecule was not in accordance with the experimental results (128). This comparison between experimental and theoretical data sets suggests that class II binding prediction tools are useful, but they have to be used with caution. Therefore, by different ways, both PvMSP-9 and PvDBP studies highlighted the combination of *in silico* analysis and the experimental confirmation as the ideal method. Therefore, actually there are accumulated evidences of successful use of bioinformatics on *P. vivax* vaccine research. For example, peptide sequences of PvRBP1 promiscuous for binding to HLA class II molecules were selected by ProPred algorithm and the IEDB server (http://www.iedb.org/) for allele binding score and population coverage. The most promising peptide sequences were included in a PvRBP-1 chimeric antigen containing the predicted promiscuous T-cell epitopes and known B-cell epitopes and presented no HLA restriction in naturally acquired immune response of exposed individuals (86). Moreover, Cespedes et al. also used the identification and selection of novel antigens by structure, binding predictions and protein motifs. A total of 50 *P. vivax* antigens were selected based on proteome and transcriptome data of *P. falciparum* orthologs. After immunological confirmation, four peptides were experimentally confirmed as truly immunogenic peptides and were preselected for further preclinical trials (129). Lastly, beyond the vaccine field, the prediction tools have also been used in a comparative analysis of epitopes from lactate dehydrogenase (LDH) protein (130). Based on *P. vivax* and *P. falciparum* LDH sequences, T-cell epitope prediction indicated that 28 HLA alleles could recognize pLDH antigen epitopes. Interestingly, despite a large number of potentially common or similar epitopes, specific Pv-LDH and Pf-LDH epitopes were also predicted and, if experimentally confirmed, could be involved in future specific diagnostic rapid tests.

**Table 3:** **Bioinformatics approaches applied to epitope selection in relation to MHC alleles**.

Program/database	Approach	HLA	*P. vivax* research aplication	Reference
SYFPEITHI	Database comprising more than 7000 endogenous peptide sequences known to bind class I and class II MHC molecules (131)	Class I and Class II	Comparative analysis of epitopes of *P. vivax* and *P. falciparum* lactate dehydrogenase (LDH) protein, based on LDH sequences	(128, 130)
			Comparison of immunologically identified universal epitopes in *Plasmodium vivax* Duffy-binding protein and *in silico* prediction results	
TEPITOPE/ProPred	Promiscuity evaluation based on virtual IC50 assay of single amino acid variants of peptide sequences (132, 133)	Class II	T-cell epitope mapping on the 33-kDa region of *P. vivax* MSP1 vaccine candidate Identification and confirmation of promiscuous epitopes in PvMSP-9	(86, 97, 134)
			Selection of promiscuous epitopes in PvRBP-1 for inclusion in a chimeric recombinant protein	
RANKPEP	Position-specific scoring matrices of known T cell epitopes (135)	Class I and Class II	Not found	–
MULTIPRED	Evaluation of potential promiscuous T cell epitopes using neural network and hidden Markov model algorithms (136)	Class I and Class II	Not found	–
NetMHC	Neural network approach to associate binding preferences and MHC sequences known T cell epitopes, MHC structures, and sequences (137)	Class I and Class II	*In silico* analysis of PvMSP-1 sequence to find potential promiscuous T CD4 and T CD8 epitopes	(126, 138)
			Identification, localization, and confirmation of MHC-restricted CD8^+^ T cell epitopes within the *Pf*AMA1 protein and *Pv*AMA1 domain III	
EpiDOCK	Converts the input sequence into a collection of overlapping non-amers and predicts binding to the 23 most frequent human MHC class II and a score is assigned (139)	Class II	Not found	–
IEDB	Immune Epitope Database Consensus method consisting of NN-align, SMM-align, MetMHCPan, and/or Combinatory Library available for the sequence (140, 141)	Class I and Class II	Identification of allele binding score of predicted promiscuous epitopes and evaluation of population coverage selected PvRBP-1 antigens	(86, 129)
			Identification and selection of potential multi-specie (*P. vivax* and *P. falciparum*) antigens by protein structure, binding predictions and protein motifs	

In summary, with the concomitant advent of whole-genome sequencing and advances in bioinformatics, the vaccinology field changed in the last few decades, providing the opportunity of describing novel antigens and improving the already known. Consequently, the focus in vaccine design shifted to explore antigens susceptible to antibody recognition and T-cell induction through comparative pan genome reverse vaccinology. Even though, in most of cases, experimental confirmation is necessary, high-accuracy predictions are available for any HLA known, non-human primates, mouse strains, and other mammals. Therefore, those “reverse immunology” systems have become highly accessible, and they can be a fast and efficient alternative when some conventional vaccinology strategies are difficult, especially when dealing with non-culturable microorganisms, as *P. vivax*.

## Concluding Remarks

The number of studies involving MHC polymorphism and *P. vivax* specific immune response and clinical outcome are still increasing, and there are several similarities and disparities among these association studies. Despite the variation of MHC genes, alleles and/or haplotypes in different clinical and epidemiological scenarios, the association between MHC genes and *P. vivax* has been demonstrated in the majority of studies presented. We believe that the inconsistency of some data may derive from the fact that a large number of potential risk factors, such as nutritional status, coinfections, and relapses, which could influence the specific immune response, are almost impossible to be controlled in malaria endemic areas. Other issues are the small sample size, the heterogeneity of human populations in different endemic areas, and of course, the complexity of MHC genes. Therefore, since population-based cohorts with a single *P. vivax* infection represent a valuable but uncommon resource for genetic studies, more sophisticated analytical approaches are needed to study the expression of MHC genes in such different exposure conditions to determine the precise role of such polymorphisms as determinant for *P. vivax* susceptibility, immune response, and its outcome in disease progression.

## Author Contributions

JL-J and LP-R wrote the review.

## Conflict of Interest Statement

The authors declare that the research was conducted in the absence of any commercial or financial relationships that could be construed as a potential conflict of interest.
